# Rolling back the prison estate: the pervasive impact of macroeconomic austerity on prisoner health in England

**DOI:** 10.1093/pubmed/fdz058

**Published:** 2019-05-24

**Authors:** Nasrul Ismail

**Affiliations:** ESRC PhD Researcher in Public Health, Centre for Public Health & Wellbeing, University of the West of England (UWE Bristol), Frenchay Campus, Coldharbour Lane, Bristol, UK

**Keywords:** austerity, England, neoliberalism, prisoner health, prisons, recession

## Abstract

Prisons offer policymakers an opportunity to address the pre-existing high prevalence of physical and mental health issues among prisoners. This notion has been widely integrated into international and national prison health policies, including the Healthy Prisons Agenda, which calls for governments to address the health needs of prisoners and safeguard their health entitlement during imprisonment, and the Sustainable Development Goals 2030 concerning reducing inequality among disadvantaged populations.

However, the implementation of the austerity policy in the United Kingdom since the re-emergence of the global financial crisis in 2008 has impeded this aspiration. This interdisciplinary paper critically evaluates the impact of austerity on prison health. The aforementioned policy has obstructed prisoners’ access to healthcare, exacerbated the degradation of their living conditions, impeded their purposeful activities and subjected them to an increasing level of violence.

This paper calls for alternatives to imprisonment, initiating a more informed economic recovery policy, and relying on transnational and national organizations to scrutinize prisoners’ entitlement to health. These systemic solutions could act as a springboard for political and policy discussions at national and international forums with regard to improving prisoners’ health and simultaneously meeting the aspirations of the Healthy Prisons Agenda and the Sustainable Development Goals.

## Introduction

The World Health Organization (WHO) established the Healthy Prisons Agenda as a global priority in 1995^1^ in response to overwhelming evidence of high levels of physical and mental health problems among prisoners.^2,3^ Approximately seven out of every 10 prisoners suffer from two or more mental disorders,^2^ with at least half diverted from prisons to mental health institutions presenting with comorbidities of mental health problems and substance addiction.^4^ One in six of the incarcerated population is ageing; this group typically exhibits complex medical needs and higher social care dependencies compared with younger prisoners.^3,5^ Prisoners serving sentences for theft, constituting 39% of female prisoners in England and Wales, are commonly affected by substance misuse, coercive relationships, financial difficulties and debt.^6,7^ Around 26% of prisoners have identified themselves as belonging to minority ethnic groups—double the number in the UK’s general population—with undercurrent themes of obesity and high blood pressure among black women and a higher rate of self-harm among South Asian women.^8,9^

The HM Inspectorate of Prisons in England and Wales adopted the Healthy Prisons Agenda and committed to the standard that health services provided in prisons should be equivalent to those available in the community and that such services should adopt the whole-prison approach.^10^ The state has charged the five national health and justice organizations that form the National Partnership Agreement for Prison Healthcare in England 2018–21 with ensuring that this equivalence-of-care principle is implemented.^11,12^

Sim and Scott contest the expectation that prisons are a health-promoting setting. First, they argue that society grants prisoners ‘less eligibility’ in terms of care, implying that they are undeserving of what the lowest social class in the free community can access.^13^ Second, they argue that institutions are hostile landscapes that systematically refuse to meet prisoners’ needs.^14^ Nonetheless, the adoption of the Healthy Prisons Agenda and the United Nations’ Sustainable Development Goals 2030, of which Goal 10 acknowledges the need to reduce inequality among disadvantaged populations, including prisoners, and to address the health needs of prisoners and others who interface with the criminal justice system,^15^ at least represents a symbolic commitment to the equivalence-of-care principle.

This interdisciplinary article seeks to examine how austerity, which re-emerged after the global financial crisis of 2008, jeopardizes the commitment towards improving prison health. Using substantive data since 2010, this work will articulate how austerity is fundamentally a political imperative rather than an economic one; it will explore the likelihood of the country breaching its international obligations on prison health, and advance systemic recommendations in order to alleviate the harmful austerity measures that endanger prisoners’ health. In so doing, it will focus on English prisons, unless otherwise stated, as eight out of ten prisons in the UK are located in England and the prisons situated in the devolved administrations of Wales, Scotland and Northern Ireland are subject to different policy responses and enforcement.^16^ However, on occasions, the statistics used here will refer to England and Wales because the state does not provide greater specifics.

## Austerity as a political choice

The insolvency of the Northern Rock Bank in 2007, the collapse of Lehman Brothers and the financial deceit in the US mortgage market combined to cause a global economic tsunami.^17^ UK bank bailouts soon followed in 2008 and the ensuing recession precipitated the adoption of austerity in the UK in 2010.^17^ The Coalition Government that took office in May 2010 promptly embraced neoliberal economics which advanced public sector spending reductions as a means of securing deficit reductions in the short term and maintaining confidence in the country’s financial stability in the long term.^18^

**Fig. 1:**
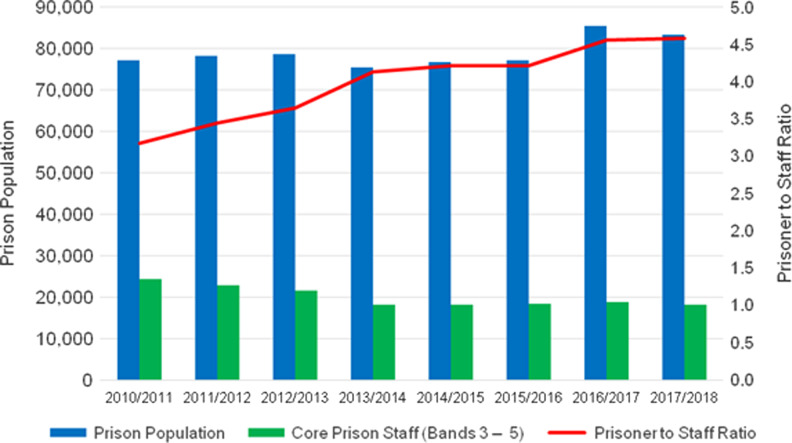
Prison population and core prison staff in England and Wales, 2010/2011 to 2016/2017. Sources: HM Prison and Probation Service, 2017, and the Ministry of Justice, 2017.

The state reduced funding for Her Majesty’s Prison and Probation Service by 22%, from £3.47 billion in 2010/2011 to £2.71 billion in 2016/17.^19^ The number of front-line prison officers in English and Welsh prisons dropped by some 30% between 2014 and 2017; most were offered either voluntary redundancy, termination of fixed-term contracts or early retirement packages.^20,21^ At the same time, the number of prisoners remained high.^22^ This created a severe staffing deficiency, with 4.6 prisoners for every staff member (Fig. 1), fewer than half of the number found in other European countries.^23^ Thus, reduced funding can be linked to the current overcrowding and instability of the carceral regime. Although prison healthcare funding by England’s National Health Service has been relatively ring-fenced at £400 million since 2013, day-to-day healthcare delivery is highly dependent upon a stable prison regime, which is deteriorating.^24^

Despite being justified as a means of reducing the state deficit, a growing body of evidence suggests that austerity was a political choice, rather than an economic imperative. Although the UK was not a member of the Eurozone, the then-Chancellor of the Exchequer George Osborne imposed severe cuts to national finances, similar to those that the Europe Commission, the European Central Bank and the International Monetary Fund (the Troika) would have imposed on Greece, Ireland and Portugal as part of their bailout conditions.^18^ Despite the government’s 2018 Budget announcement that ‘the era of austerity [is] finally coming to an end’,^25^ austerity shows no sign of abating. Considering the abundance of economic evidence of its failure, its continued implementation signifies a political choice rather than a practical necessity.

The mass socialization of state debt through the austerity programme, along with the selective distribution of scarce taxpayer money, breeds an individualistic mindset. Politicians frame health and social care as individual choices rather than collective responsibilities, and thereby justify withdrawing state support.^26^ Such an approach leaves prisoners in a precarious situation as they have no alternative but to depend on state provisions. Several recent studies have demonstrated that the adoption of austerity measures has adversely affected healthcare access for marginalized populations throughout the UK, particularly in systems with low levels of social protection.^27,28^ As such, individualism serves as an artificial barrier that hinders the state from engaging in structural interventions that would improve the lives of prisoners, which simultaneously weakens the link of altruism within society and withdraws the safety blanket that once protected prisoners.

## Potential breach of international agreements that protect prisoners’ entitlement to health

Prisons are places of punishment. They become places of double punishment when they imperil prisoners’ health, and austerity measures represent a significant peril. The fragmentation of access to healthcare, poor living conditions, barriers to participation in purposeful activities and an increasing level of violence under austerity exacerbate the existing burden of ill-health among prisoners. While a time-lag effect has delayed the negative impact of austerity on prisoner health, severe prison staffing issues in the majority of English prisons have emerged over the years as the epicentre of the unstable prison regime.

### Impeding access to healthcare

Despite the level of financial investment in prison healthcare, alongside initiatives for smoke-free prisons, opt-out testing for blood-borne pathogens and the integration of mental health and substance misuse services,^29–31^ the regular cancellation of hospital appointments and an increase in waiting times to access treatment have mediated reformist aspirations. On average, prisoners missed 15% of medical appointments, predominantly due to a lack of discipline officers to escort them.^32^ Additionally, in 2017, two-thirds of prisoners who required treatment for acute mental health problems waited longer than 14 days to be seen, and some waited over a year to be transferred to a secure hospital.^32^ In parallel with the issue of the recruitment and retention of prison staff, evidence demonstrates that prison healthcare systems suffer from understaffing issues which hampers the delivery of the prison health agenda.^33,34^

The inadequate healthcare support in English prisons runs the risk of breaching Article 3 of the European Convention of Human Rights, which bans torture and inhuman or degrading treatment or punishment. In the two cases in which a complainant accused the UK of violating Article 3 – McGlinchey v United Kingdom^35^ and Price v United Kingdom^36^ – the court held that the state had a positive obligation to protect the health and wellbeing of detainees, particularly when a prisoner is at increased vulnerability following severe health concerns, as is increasingly prevalent in English prisons. Article 3 denotes absolute rights, which the government can only transgress in times of war or other public emergencies. The principles from McGlinchey and Price are binding upon national authorities, and prisoners’ progressively deteriorating access to healthcare clearly violates these obligations.

### Degrading living conditions

The growth in the prison population in England has exceeded the increase in the general population by 10% since 2010, leading to prison overcrowding as funding for new facilities has not kept pace with this trend.^37^ Along with lengthy confinement in poorly maintained cells and dilapidated facilities, and the psychologically degrading conditions for long-term prisoners,^38^ the unhygienic living environment can accelerate the progression of disease, an inhumane and degrading aspect of the current prison regime. In fact, as early as 1990, the European Committee for the Prevention of Torture and Inhuman or Degrading Treatment or Punishment (CPT) highlighted the problem of overcrowding in English prisons, and austerity has only served to intensify this issue.^20^ A supportive and stable environment is necessary to establish prisoner health,^39^ but England provides a poor physical environment.

Prisoners in HMPs Doncaster and Pentonville suffered from overcapacity, 152% (*n* = 1122) and 141% (*n* = 1277) beyond the certified normal accommodation rates of 738 and 906 respectively, as well as unsanitary cells.^20^ These conditions included pest and vermin infestations and dilapidated bathroom facilities, and, due to inadequate funding, there were no plans for refurbishment.^38^ In most cases, inmates spent up to 22 hours a day locked in a small cell, where they ate all their meals, with a poorly screened or unscreened lavatory just inches from their bed and food.^38^ Based on cases in which Article 3 was applied elsewhere—Dougoz v Greece,^40^ Peers v Greece^41^ and Kehayov v Bulgaria^42^—these conditions may have also violated Article 3. The point of law applied in Karalevicius v Lithuania^43^ and Staykov v Bulgaria^44^ stipulates that a lack of financial resources does not absolve the state from its obligations to protect prisoners from inhuman or degrading treatment.

The government responds to the calamitous situation in English prisons only when it coheres with its neoliberal vision. Whitehall has unveiled a plan to build enough prisons to house 10 000 new prisoners.^45^ These super-prisons will not have sufficient accommodation to meet the health needs of detainees and they are unlikely to ease the overcrowding and degrading living conditions in English prisons; indeed, evidence suggests that building new prisons merely swells the prison population.^46^

### Lack of access to purposeful activities

**Fig. 2:**
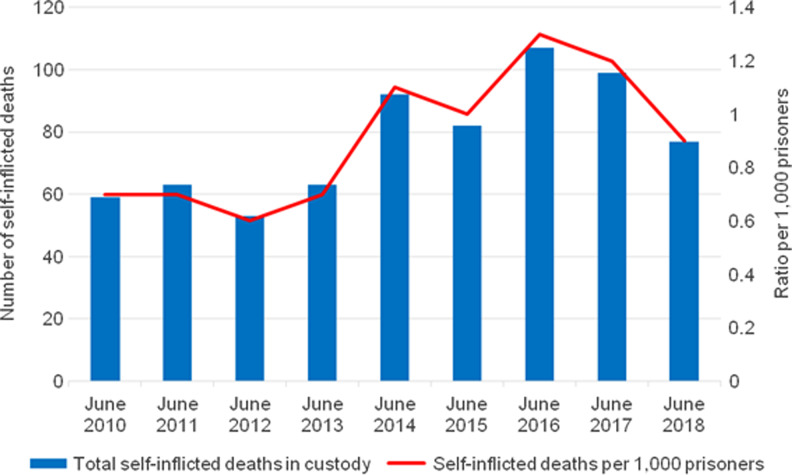
Number of self-inflicted deaths in prisons in England and Wales, June 2010 to June 2018. Source: the Ministry of Justice, 2018.

**Fig. 3:**
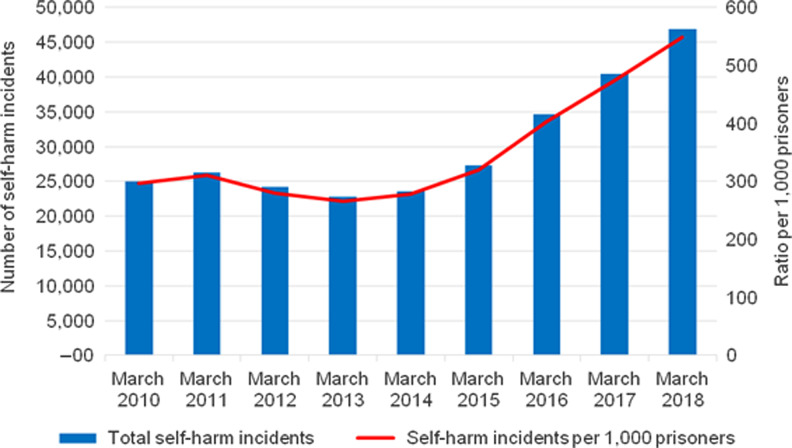
Number of self-harm incidents in prisons in England and Wales, March 2010 to March 2018. Source: the Ministry of Justice, 2018.

Staff shortages have been linked to limitations on prisoners’ entitlement to access to purposeful activities. Despite the expectation of HMI Prisons that prisoners should move freely for at least 10 hours a day, only 17% of prisoners did so in 2018.^38^ Moreover, inspections conducted at HMPs Winchester, Wormwood Scrubs and Pentonville revealed that their combined population of 2788 prisoners spent up to 22 hours a day in their cells without access to educational or social activities.^20^ The lack of access to purposeful activities not only represents an inhumane condition, it can also progressively harm mental health, within what is considered to be a confined, excluded and controlled setting.^47^ The 23% increase in suicides among prisoners between June 2010 and June 2018 (Fig. 2), as well as the 47% increase in self-harm between March 2010 and March 2018, associated with the use of *Spice* (a synthetic cannabinoid), which continued to increase in the subsequent months (Fig. 3), are reasons for this type of harm being so prevalent.^48,49^ The lack of purposeful activities provides a useful context for understanding the trend behind the drug-seeking, drug-taking and risk-taking behaviour that contributes to the increasing level of self-harm and suicides.^38,49^

Women account for 5% of the total prison population but 19% of self-harm incidents among prisoners.^48^ Addressing these risks will require improving the healthcare provision and access to purposeful activities, providing more support to manage emotional resilience and accountability for implementing recommendations, and post-death investigations and inquests.^50^

### Increasing levels of violence

**Fig. 4:**
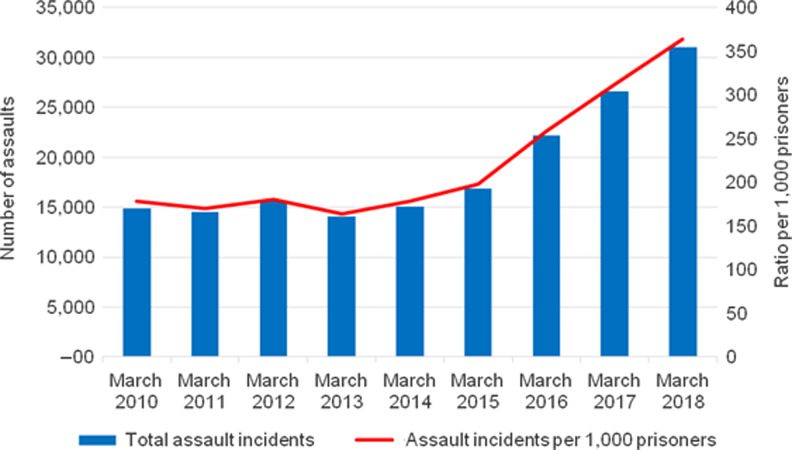
Number of assault incidents in prisons in England and Wales, March 2010 to March 2018. Source: the Ministry of Justice, 2018.

Assaults in prisons have increased since 2010 (Fig. 4); by the end of March 2018, there had been 31 025 recorded incidents of assault, including both prisoner-on-prisoner and prisoner-on-staff cases.^48^ This represents a 52% increase since March 2010.^48^ The deployment of tactical intervention teams from the National Tactical Response Group, which responded to 30–40 hostage-taking and concerted riot incidents per month in 2015, similarly reflects the increased violence.^51^ The underreporting of incidents of violence, both in terms of their number and severity, has led to an inaccurate picture of the severity of the situation in English prisons.^20^ Equally, there has been an increased use of force on prisoners, as well as significant gaps in corresponding governance.^38^

In response to the current level of instability, a concerted effort has been made to reduce the turnover of prison staff. In 2017, the state pledged an additional £13 million in ‘emergency funding’ for English and Welsh prisons in response to CPT claims that underfunding violated national law.^20^ Although this translated into an increase of 3111 prison officers between 2016 and 2018,^52^ improvements may take some time to materialize. Furthermore, the urgent notification protocol triggered by the Chief Inspector of Prisons to the Secretary of State regarding his grave concerns about the treatment of, and conditions for, prisoners at HMPs Nottingham, Exeter, Birmingham and Bedford underscored the cumulative effect of inexperienced staff, poor supervision and lack of accountability concerning the use of force towards prisoners.^53^ Moreover, it also reflected delays and apathy in response to prisoners’ complaints about incidents of violence, thus fuelling an atmosphere of fear and a lack of confidence in the staff and prison management to maintain institutional safety.^53^

## Proposals to overturn harmful austerity measures

The state must make a renewed commitment to the Healthy Prisons Agenda if it is to comply with international law and fulfil the expectations outlined by Sustainable Development Goal 10. There are three possible scenarios for meeting these obligations.

First, considering that the annual cost per prison place is £38 042^54^ the state should consider alternatives to imprisonment. Fines, community service and restorative solutions are more proportionate, more responsive to prisoners’ needs and less disruptive to prisoners’ families and social networks, especially for women and those with mental health issues.^47,50^ In fact, other Western societies have realized that they are no longer able to financially support continued penal expansion, prompting them to consider less expensive, more humane and sustainable forms of correction.^55,56^

Diverting prisoners with acute mental health problems to hospital or community-based treatment,^32^ together with utilizing the Home Detention Curfew, abolishing sentences of imprisonment of up to 12 months for nonviolent offenders and limiting the proliferation of laws establishing new offences,^57^ will ensure that prisons house only those whose incarceration protects public safety, thus allowing prison healthcare resources to be concentrated on a smaller cohort of users. Recent political interest in reducing short-term jail sentences can be used as leverage to encourage policymakers to consider alternatives to detention, and such a reformation of sentencing practices can reduce the need for prison institutions.^58,59^

Second, a more informed economic recovery policy that addresses the actual root cause of the fiscal crisis, such as reinforcing more progressive taxation among multinational corporations, tackling tax avoidance and evasion, and addressing the discrepancies in corporate tax rates,^60,61^ would be compatible with increased spending on prisoner health. For instance, a negligible recovery rate of 0.05% (five pence per pound sterling) for the £5.8 billion tax evasion by multinational companies^62^ could raise £29 million. Beyond addressing funding shortages in the prison regime, such funds could be allocated towards community-based services that are rooted in welfare, health, and social care services, arguably providing a better alternative to imprisonment.^50^ Iceland, Germany and the US, countries which all opted for fiscal stimulus rather than austerity in response to the global financial crisis, recorded better measures of mental health and a reduction in suicides among prisoners and the general population, as well as accelerated economic recovery.^63^ Whitehall should increase investment in prisons, and moving beyond Gross Domestic Product (GDP) as the measure of societal growth is a vital aspect of the much-needed paradigm shift.

Finally, transnational organizations, such as the Troika and WHO, and non-governmental organizations such as Amnesty International and the International Committee of the Red Cross, can provide an antidote to the democratic deficit in global governance.^64,65^ Their supranational status should enable them to articulate the impact of austerity on prisoners, reminding states that health standards should not be breached, whilst monitoring compliance and, as a last resort, naming and shaming human rights violators. In order to prevent future economic crises from imperilling human rights, international organizations must address the disconnect between the preservation of human rights and the focus of key international players on economic performance. National advocacy organizations, supported by the lived experiences of prisoners who are capable of articulating the pains of imprisonment during austere times, and the campaign to reform the existing sentencing policy to reduce the number of prisoners, can support this progressive movement from the bottom up.^14,50^

## A call for action

This is the first interdisciplinary research paper to analyse the pervasive impact of the government’s austerity measures on prisoners’ health in England. The political rhetoric of austerity prioritizes individualistic thinking and dismantles the welfare state by framing the healthcare needs of prisoners as individual deficits. Prisoners suffer from degrading living conditions and a lack of access to healthcare, in addition to being denied access to purposeful activities, while enduring violence on a daily basis. Under these circumstances, austerity perpetuates ongoing instability in English prisons, hampering progress towards the Healthy Prisons Agenda and Sustainable Development Goal 10, and increasing the likelihood of violation of the European Convention of Human Rights principles.

To address its systemic failure, the state must consider alternatives to imprisonment and initiate a more informed economic recovery policy, whilst relying upon transnational and national advocacy organizations to scrutinize prisoners’ entitlement to health. The findings herein have significant relevance beyond England, particularly for other jurisdictions that have adopted austerity in relation to prisons following the 2008 global economic recession, thereby providing further opportunities for comparative research on these important and as yet understudied phenomena.

While the current evidence problematises the mechanisms by which the government mobilizes austerity measures across English prisons, it has yet to contextualize the impact of austerity on the availability, accessibility, acceptability and quality of health services in prisons. In addition, the potential legal and policy risks of the continued approach of austerity in terms of prison health, along with mitigation strategies, require further unpacking. This article therefore calls for studies to address these gaps, to provide a narrative that calls for fundamental reform, from a destructive system that encourages state oppressiveness to a more positive and inclusive system, so that England might live up to its own self-concept as a progressive society.

## Licence to publish

The author grants an exclusive licence to the Faculty of Public Health of the Royal Colleges of Physicians of the UK.
